# Boosting ridge for the extreme learning machine globally optimised for classification and regression problems

**DOI:** 10.1038/s41598-023-38948-3

**Published:** 2023-07-21

**Authors:** Carlos Peralez-González, Javier Pérez-Rodríguez, Antonio M. Durán-Rosal

**Affiliations:** Department of Quantitative Methods, Universidad Loyola Andalucía, Córdoba, Spain

**Keywords:** Computational science, Computer science, Information technology, Scientific data

## Abstract

This paper explores the boosting ridge (BR) framework in the extreme learning machine (ELM) community and presents a novel model that trains the base learners as a global ensemble. In the context of Extreme Learning Machine single-hidden-layer networks, the nodes in the hidden layer are preconfigured before training, and the optimisation is performed on the weights in the output layer. The previous implementation of the BR ensemble with ELM (BRELM) as base learners fix the nodes in the hidden layer for all the ELMs. The ensemble learning method generates different output layer coefficients by reducing the residual error of the ensemble sequentially as more base learners are added to the ensemble. As in other ensemble methodologies, base learners are selected until fulfilling ensemble criteria such as size or performance. This paper proposes a global learning method in the BR framework, where base learners are not added step by step, but all are calculated in a single step looking for ensemble performance. This method considers (i) the configurations of the hidden layer are different for each base learner, (ii) the base learners are optimised all at once, not sequentially, thus avoiding saturation, and (iii) the ensemble methodology does not have the disadvantage of working with strong classifiers. Various regression and classification benchmark datasets have been selected to compare this method with the original BRELM implementation and other state-of-the-art algorithms. Particularly, 71 datasets for classification and 52 for regression, have been considered using different metrics and analysing different characteristics of the datasets, such as the size, the number of classes or the imbalanced nature of them. Statistical tests indicate the superiority of the proposed method in both regression and classification problems in all experimental scenarios.

## Introduction

In the last decade, Extreme Learning Machine (ELM)^1^ has become a popular methodology in Machine Learning challenging problems, for instance, brain-computer interfaces^2^, the prediction of the remaining rolling bearing useful life^3^, the origin detection of fennel which is of great importance in food flavouring^4^, the COVID-19-pneumonia prediction^5^, EGG classification for brain-computer interface^6^, water network management^7^, and wheat yield prediction^8^, among others. ELM theories claim that the hidden layer parameters, that is, the weight and bias in single-hidden layer feed-forward networks, do not need to be tuned, but they can be generated randomly, independently of the training dataset^9^. Thus, only the output weights are computed in a single step by employing the least-squares estimated solution. Due to this random initialisation, ELM training speed is more efficient compared to the traditional solvers for neural networks, for instance, those based on back-propagation^10,11^, without losing performance, and even improving it.

One of the drawbacks of ELM models is that it requires a high number of neurons for the hidden layer because the nonlinear combination of features is explored randomly^12^. Due to this, several methods have been investigated for reducing this randomness without increasing the computation time or the algorithm’s complexity, such as pruning^13^, swarm optimisation^14,15^ and ensemble learning methods.

In this context, several ensemble methods for ELM models have been proposed, e.g., ensembles for regression^16^, fuzzy ensembles for big data classification^17^, deep ensembles for time series forecasting^18^, incremental Meta-ELM with error feedback^19^ or weighted kernel ELM ensembles for imbalanced datasets^20^. Furthermore, many ELM ensemble methods have been applied to real-world problems, such as the prediction of ocean wave height^21^, human activity recognition^22^, calibration of near-infrared spectroscopy^23^ or birdsong recognition^24^. In general, ensembles aim to improve the generalisation error using a mixture of classifiers or regressors, known as base learners in the ensemble learning framework. The performance improvement is associated with diversity among the base predictors, i.e. it is essential for the generalisation of the ensemble that the base learners disagree as much as possible^25^. There are many ways to combine individual predictions. Thus several voting methods have been proposed to improve the efficiency of these ensembles, such as Bagging^26^, Boosting^27^, incremental learning system using local linear experts^28^ or a variation of Boosting constructed from a functional gradient descent algorithm with the L2-loss function^29^, among others. The ensemble methodologies known as Bagging and Boosting are the most widely used approaches, mainly because of their ease of application and their ensemble performance^30^. The key to these ensemble methodologies lies in the training data to generate diversity. In this way, diverse solutions to the optimisation problem associated with the base predictors are implicitly sought through data sampling^31^.

Specifically, in the field of Boosting philosophy, a particularly interesting algorithm is Boosting Ridge (BR)^32^. This ensemble algorithm, designed originally for regression problems, trains the base learners sequentially, setting the residual of the previous predictor as the training target. The first base learner is the predictor for the original target. Subsequently, the error between the prediction on the training set and the target is calculated, and this residual is the new target. The second predictor is trained with this residual. After calculating the error between the second predictor and the first residual, a third residual is calculated, which is the target of the next predictor. The process is repeated until the number of base learners is reached. BR shows its importance in many applications, such as early-stage breast cancer detection^33^, microarray survival models^34^ and criminal recidivism predictions^35^.

The addition of base learners does not continuously improve the ensemble since there is a trade-off between diversity among the base learners and the final ensemble performance^36^. Furthermore, in the boosting methodology, although each base learner is added to reduce the error of the previous ones, the saturation of the base learners eventually appears. The saturation occurs when the ensemble cannot improve the generalisation error despite introducing more and more base learners. Also, if the number of base learners is fixed, saturation or even overfitting could be produced because the base learners become stronger (more accurate). It is given that increasing the number of hidden neurons reduces the diversity in the ensemble^37^, which is needed to improve the ensemble performance^25^. To overcome the saturation and to give an approach model selection,^38^ proposes the use of genetic algorithms to select the optimal number of base learners involved in the ensemble,^39^ proposes an adaptative stopping rule via adjusting the regularisation parameter, and^40^ relies on diversity measures to establish the upper bound of a number of base learners.

Like other ensemble methodologies^36,41^, BR aims to train each base predictor separately and then combine their results. BR algorithm for ELM-based learners (BRELM) was initially proposed by Ran et al.^42^. With this background and to overcome the main drawbacks mentioned above, this paper proposes a new boosting algorithm that removes the need to add base learners sequentially, leading to saturation. Also, using strong instead of weak base classifiers does not worsen the ensemble’s performance. For this, several predictors are optimised at once to calculate the optimised ensemble parameters globally. The formulation of the error function allows the development of an analytical solution for the parameters of the ELM-based learners to find the weights of the output layers for each base learner in a single step. Moreover, this ensemble learning method achieves better results than the sequential BR, as the error is optimised globally in the ensemble and not for each base learner.

Summarising, the novel contributions of this work are:

- The optimisation of the weights of the output layer of a Boosting Ridge for Extreme Learning Machine ensemble in a single step instead of iteratively, with the objective of reducing the generalisation error.
- The use of different input layers mappings with different parameters for their hidden layers, made possible by the new optimisation approach resulting in the so-called Generalised Global BRELM (GGBRELM), tends to a better diversity of the ensemble.
- Avoid the problem of ensemble saturation and overtraining by making the new proposal work well when the base classifiers become stronger. For example, it is known that by increasing the number of neurons in the ELM networks of the base learners, each one achieves good performance, but, in return, the ensemble’s performance is reduced. With the new proposal, this problem is solved.
- The application of the methodology to more than 120 classification and regression datasets from different domains shows that the proposal works better than the state-of-the-art methods and can be applied to any real-world problem.
- The performance of the proposed methodology analysis considers different dataset properties such as size, number of classes or imbalance.

This paper is organised as follows: “State-of-the-art algorithms” Section summarises the notation and formulation of the ELM, BR and BRELM algorithms. “Methodology of the proposal” Section develops the proposed methodology about the globalisation of BRELM and its generalised version GGBRELM, shows a graphical comparison of the methodologies, and includes an analysis of their computational costs. The experimental design is set in Section experimental design, while “Discussion of the results” Section explains the most highlighted results, including statistical analysis. Finally, “Conclusions” Section collects the main conclusions obtained in the work.

## State-of-the-art algorithms

This section introduces the notation and formulation of the two algorithms on which this proposal is based, i.e., ELM predictor and BR ensemble methodology.

### Extreme learning machine

For a simple supervised learning problem, dataset ${\mathscr {D}} = \{ ({\textbf{x}}_1, {\textbf{y}}_1), \ldots , ({\textbf{x}}_n, {\textbf{y}}_n ),$ $\ldots , ({\textbf{x}}_N, {\textbf{y}}_N) \} = \{ ({\textbf{x}}_n, {\textbf{y}}_n) \}_{n=1}^N$ consists in a set of *N* patterns, each one with a vector of features, ${\textbf{x}}_n$ and target associated, ${\textbf{y}}_n$.

- ${\textbf{x}}_n \in {\mathbb {R}}^K$ is the data information for the *n*-th pattern, where *K* is the number of input variables.
- ${\textbf{y}}_n$ is the target variable for the *n*-th pattern. In case of regression problems, ${y}_n \in {\mathbb {R}}$ since it is a number. In classification problems with *J* classes, the target can be expressed as “1-of-*J*” encoding, ${\textbf{y}}_n \in {\mathbb {R}}^J$. Each component *j* of ${\textbf{y}}_n$ is $y_{j, n} = 1$ if *n*-th pattern belongs to class *j* and $y_{j, n} = 0$ otherwise.

Using “1-of-*J*” encoding, a classification can be rewritten as a multi-regression problem. Thus, ELM model is explained for regression problems in this subsection, and the explanation for classification is summed up at the end. A predictor $f: {\mathbb {R}}^K \rightarrow {\mathbb {R}}$ inferring a function that maps an input *n*-th pattern ${\textbf{x}}_n$ to an output target ${y}_n$, using relationships from labeled dataset ${\mathscr {D}} = \{ ({\textbf{x}}_n, {y}_n) \}_{n=1}^N$. In particular, Extreme Learning Machine (ELM) model build this function:

1 $$\begin{aligned} f ({\textbf{x}}) = {\textbf{h}}'({\textbf{x}}) \varvec{\beta }, \end{aligned}$$

where:

- ${\textbf{h}}: {\mathbb {R}}^K \rightarrow {\mathbb {R}}^D$ is a non-linear mapping of the input layer. It transforms the pattern ${\textbf{x}}_n$ from the original feature space ${\mathbb {R}}^K$ to the transformed space ${\mathbb {R}}^D$, where *D* is the number of neurons in the hidden layer. This mapping is explicitly computed as
  2 $$\begin{aligned} {\textbf{h}}({\textbf{x}}) = (\phi _d ({\textbf{x}};{\textbf{w}}_{d},b_{d}), \, d = 1, \ldots , D), \end{aligned}$$
  with $\phi : {\mathbb {R}}^K \rightarrow {\mathbb {R}}$ as the activation function for the neuron *d*, and the weights ${\textbf{w}}_{d}$ and biases $b_{d}$ are randomly generated.
- $\varvec{\beta }: {\mathbb {R}}^{D}$ is the vector of weights in the output layer, that are found in the optimisation problem:
  3 $$\begin{aligned} \min _{\varvec{\beta } \in {\mathbb {R}} ^{D}}\ \Vert {\textbf{H}}\varvec{\beta }-{\textbf{Y}} \Vert ^2 + C \Vert \varvec{\beta } \Vert ^2, \end{aligned}$$
  where ${\textbf{H}} = \left( {\textbf{h}}' \left( {\textbf{x}}_{1}\right) , \ldots , {\textbf{h}}' \left( {\textbf{x}}_{N}\right) \right) \in {\mathbb {R}}^{N \times D}$ is the output of the hidden layer for the training patterns, $${\textbf{Y}} = \left( {\begin{array}{c} {\textbf{y}}_{1} \\ \vdots \\ {\textbf{y}}_{N}\\ \end{array} } \right) \in {\mathbb {R}}^{N}$$ is the matrix with the desired targets and $C > 0$ is an user-specified term, that controls the regularisation in the model^12^.

Equation (3) represents a convex minimisation problem with error and regularisation terms. The error term ${\Vert {\textbf{H}} \varvec{\beta } - {\textbf{Y}} \Vert }^2$ adjusts the coefficient vector $\varvec{\beta }$ in order to minimise the error of the prediction ${\textbf{Y}}$, while the regularisation term ${\Vert \varvec{\beta }_j \Vert }^2$ is included to avoid over-fitting in the model^43^.

The optimal solution for the model is the minimum of the convex objective function in Eq. (3), and it is obtained by deriving and equaling to 0:

4 $$\begin{aligned} {\varvec{\beta }} = \left( {\textbf{H}}'{\textbf{H}} + {C} {\textbf{I}} \right) ^{-1}{\textbf{H}}'{\textbf{Y}}. \end{aligned}$$

For a classification problem, there are *J* minimisation problems as Eq. (3). The predicted class corresponds to the vector component with the highest value, that is

5 $$\begin{aligned} \arg \max _{j=1,\ldots ,J} ({\textbf{h}}({\textbf{x}}) \varvec{\beta }_{1}, \ldots , {\textbf{h}}({\textbf{x}}) \varvec{\beta }_{j}, \ldots , {\textbf{h}}({\textbf{x}}) \varvec{\beta }_{J}) \end{aligned}$$

### Boosting ridge regression (linear model)

From a linear regression model,

6 $$\begin{aligned} f({\textbf{x}}) = {\textbf{x}}' \varvec{\beta }, \end{aligned}$$

and its associated minimisation problem

7 $$\begin{aligned} \min _{\varvec{\beta }} {\Vert {\textbf{X}} \varvec{\beta } - {\textbf{Y}} \Vert }^2 + C {\Vert \varvec{\beta } \Vert }^2, \end{aligned}$$

with $${\textbf{X}} = \begin{pmatrix} {\textbf{x}}_1^{'} \\ \vdots \\ {\textbf{x}}_N^{'} \end{pmatrix} \in {\mathbb {R}}^{N \times K}$$, Tutz et al.^32^ proposed BR Regression as ensemble learning method that reduces sequentially the residual of the ensemble prediction,

8 $$\begin{aligned} \min _{\varvec{\beta }^{(1)}} {\Vert {\textbf{X}} \varvec{\beta }^{(1)} - {\textbf{Y}} \Vert }^2 + C {\Vert \varvec{\beta }^{(1)} \Vert }^2;&\,&\mathbf {\mu }_1 = {\textbf{Y}} - {\textbf{X}} \varvec{\beta }^{(1)}, \nonumber \\ \min _{\varvec{\beta }^{(2)}} {\Vert {\textbf{X}} \varvec{\beta }^{(2)} - \mathbf {\mu }_1 \Vert }^2 + C {\Vert \varvec{\beta }^{(2)} \Vert }^2;&\,&\mathbf {\mu }_2 = \mathbf {\mu }_1 - {\textbf{X}} \varvec{\beta }^{(2)}, \nonumber \\ \min _{\varvec{\beta }^{(3)}} {\Vert {\textbf{X}} \varvec{\beta }^{(3)} - \mathbf {\mu }_2 \Vert }^2 + C {\Vert \varvec{\beta }^{(3)} \Vert }^2;&\,&\mathbf {\mu }_3 = \mathbf {\mu }_2 - {\textbf{X}} \varvec{\beta }^{(3)}, \nonumber \\&\vdots&\end{aligned}$$

For an ensemble with *S* base learners, the prediction of the BR Regression model is

9 $$\begin{aligned} f ({\textbf{x}}) = \sum _{s=1}^{S} {\textbf{x}}' \varvec{\beta }^{(s)} = {\textbf{x}}' \sum _{s=1}^{S} \varvec{\beta }^{(s)}. \end{aligned}$$

### Boosting ridge extreme learning machine

BR ensemble learning methodology was adapted to the ELM community by Ran et al.^42^. The prediction of this sequential ensemble, BRELM, of *S* base learners is the following linear combination:

10 $$\begin{aligned} f ({\textbf{x}}) = \sum _{s=1}^{S} f^{(s)} ({\textbf{x}}) = {\textbf{h}}' ({\textbf{x}}) \sum _{s=1}^S \varvec{\beta }^{(s)}. \end{aligned}$$

The first base learner $s = 1$ is the standard ELM solution from Eq. (3). Later, the *s*-th base learner training stage uses all the data, but the target $\mathbf {\mu }^{(s)}$ is the residual of the previous base learner predictions,

11 $$\begin{aligned} \mathbf {\mu }^{(s)} = {\textbf{Y}} - \sum _{s'=1}^{s-1} {\textbf{H}} \varvec{\beta }^{(s')}. \end{aligned}$$

Therefore, the minimisation problem of the *s*-th base learner is

12 $$\begin{aligned} \min _{\varvec{\beta }^{(s)}} {\Vert {\textbf{H}} \varvec{\beta }^{(s)} - \mathbf {\mu }^{(s)} \Vert }^2 + C {\Vert \varvec{\beta }^{(s)} \Vert }^2, \end{aligned}$$

and the solution for the output layer of the *s*-th base learner is

13 $$\begin{aligned} \varvec{\beta }^{(s)} = ({\textbf{H}}' {\textbf{H}} + C {\textbf{I}} )^{-1} {\textbf{H}}' \left( {\textbf{Y}} - {\textbf{H}} \sum _{s'=1}^{s - 1} \varvec{\beta }^{(s')} \right) . \end{aligned}$$

## Methodology of the proposal

In this section, the Globalisation of the BRELM is proposed, along with an enhanced version called Generalised Global BRELM (GGBRELM). A methodological graphical comparison is also included. And finally, a theoretical analysis of the methodologies’ computational complexities is discussed.

The main hypothesis of this work is that the methodology based on the optimisation of all the base learners in a single step will improve the generalisation error of the ensemble. Thus, considering that this procedure will avoid the saturation of the ensemble, and therefore, for a high number of neurons (strong ELM base learners), the ensemble performance will not be reduced. Besides, the use of different input layer weights and, therefore, different mapping functions (${\textbf{h}}^{(s)}$) between the different base predictors will lead to more diversity in the ensemble.

### Global boosting ridge for extreme learning machine

The main idea behind BRELM is to reduce sequentially the error produced by the ensemble. This proposal, Global BRELM, presents the problem for each *s*-th base learner as the error reduction of the other base learners of the ensemble.

14 $$\begin{aligned}{} & {} f ( {\textbf{x}} ) = \sum _{s=1}^S f^{(s)} ({\textbf{x}}), \end{aligned}$$

15 $$\begin{aligned}{} & {} \min _{\varvec{\beta }^{(s)}} {\Vert {\textbf{H}} \varvec{\beta }^{(s)} - ( {\textbf{Y}} - \sum _{s' \ne s}^S {\textbf{H}} \varvec{\beta }^{(s')} ) \Vert }^2 + C {\Vert \varvec{\beta }^{(s)} \Vert }^2. \end{aligned}$$

Deriving respect with $\varvec{\beta }^{(s)}$ and equal to 0, some terms depend on $\varvec{\beta }^{(s)}$ while other ones depend on $\varvec{\beta }^{(s')}$, $s' = 1, \ldots , S$, $s' \ne s$,

16 $$\begin{aligned} \left( {\textbf{H}}' {\textbf{H}} + C {\textbf{I}} \right) \varvec{\beta }^{(s)} + {\textbf{H}}' \sum _{s' \ne s}^S {\textbf{H}} \varvec{\beta }^{(s')} - {\textbf{H}}' {\textbf{Y}} = 0. \end{aligned}$$

From the previous equation, a system of equations can be set up,

17 $$\begin{aligned} \begin{pmatrix} {\textbf{H}}' {\textbf{H}} + C {\textbf{I}} &{} {\textbf{H}}' {\textbf{H}} &{} \ldots &{} {\textbf{H}}' {\textbf{H}} \\ {\textbf{H}}' {\textbf{H}} &{} {\textbf{H}}' {\textbf{H}} + C {\textbf{I}} &{} \ldots &{} {\textbf{H}}' {\textbf{H}} \\ \vdots &{} \vdots &{} \vdots &{} \vdots \\ {\textbf{H}}' {\textbf{H}} &{} {\textbf{H}}' {\textbf{H}} &{} \ldots &{} {\textbf{H}}' {\textbf{H}} + C {\textbf{I}} \end{pmatrix} \begin{pmatrix} \varvec{\beta }^{(1)} \\ \varvec{\beta }^{(2)} \\ \vdots \\ \varvec{\beta }^{(s)} \end{pmatrix} = \begin{pmatrix} {\textbf{H}}' {\textbf{Y}} \\ {\textbf{H}}' {\textbf{Y}} \\ \vdots \\ {\textbf{H}}' {\textbf{Y}} \end{pmatrix}, \end{aligned}$$

so the solution to Eq. (17) can be computed just by inverting a matrix,

18 $$\begin{aligned} \begin{pmatrix} \varvec{\beta }^{(1)} \\ \varvec{\beta }^{(2)} \\ \vdots \\ \varvec{\beta }^{(s)} \end{pmatrix} = {\begin{pmatrix} {\textbf{H}}' {\textbf{H}} + C {\textbf{I}} &{} {\textbf{H}}' {\textbf{H}} &{} \ldots &{} {\textbf{H}}' {\textbf{H}} \\ {\textbf{H}}' {\textbf{H}} &{} {\textbf{H}}' {\textbf{H}} + C {\textbf{I}} &{} \ldots &{} {\textbf{H}}' {\textbf{H}} \\ \vdots &{} \vdots &{} \vdots &{} \vdots \\ {\textbf{H}}' {\textbf{H}} &{} {\textbf{H}}' {\textbf{H}} &{} \ldots &{} {\textbf{H}}' {\textbf{H}} + C {\textbf{I}} \end{pmatrix}}^{-1} \begin{pmatrix} {\textbf{H}}' {\textbf{Y}} \\ {\textbf{H}}' {\textbf{Y}} \\ \vdots \\ {\textbf{H}}' {\textbf{Y}} \end{pmatrix}. \end{aligned}$$

This solution also works for simple BR with lineal regressors, replacing ${\textbf{H}}' {\textbf{H}}$ and ${\textbf{H}}' {\textbf{Y}}$ for ${\textbf{X}}' {\textbf{X}}$ and ${\textbf{X}}' {\textbf{Y}}$ respectively.

### Generalised global boosting ridge ELM

The generalisation is as simple as making ${\textbf{H}}$ different for each *s*-th base learner,

19 $$\begin{aligned} \begin{pmatrix} \varvec{\beta }^{(1)} \\ \varvec{\beta }^{(2)} \\ \vdots \\ \varvec{\beta }^{(s)} \end{pmatrix} = {\begin{pmatrix} {\textbf{H}}^{(1)'} {\textbf{H}}^{(1)} + C {\textbf{I}} &{} {\textbf{H}}^{(1)'} {\textbf{H}}^{(2)} &{} \ldots &{} {\textbf{H}}^{(1)'} {\textbf{H}}^{(S)} \\ {\textbf{H}}^{(2)'} {\textbf{H}}^{(1)} &{} {\textbf{H}}^{(2)'} {\textbf{H}}^{(1)} + C {\textbf{I}} &{} \ldots &{} {\textbf{H}}^{(2)'} {\textbf{H}}^{(S)} \\ \vdots &{} \vdots &{} \vdots &{} \vdots \\ {\textbf{H}}^{(S)'} {\textbf{H}}^{(1)} &{} {\textbf{H}}^{(S)'} {\textbf{H}}^{(2)} &{} \ldots &{} {\textbf{H}}^{(S)'} {\textbf{H}}^{(S)} + C {\textbf{I}} \end{pmatrix}}^{-1} \begin{pmatrix} {\textbf{H}}^{(1)'} {\textbf{Y}} \\ {\textbf{H}}^{(2)'} {\textbf{Y}} \\ \vdots \\ {\textbf{H}}^{(S)'} {\textbf{Y}} \end{pmatrix}. \end{aligned}$$

The different nonlinear feature mappings in ${\textbf{H}}^{(s)}$ can be generated by any ELM method: randomisation^12^, PCA with different subsets of the training dataset^44^, elements in a pseudorandom sequence^45^, $\ldots$ As mentioned before, with this generalisation, several random weights and biases have been selected for each mapping function ${\textbf{h}}^{(s)}$ mappings, thus generating different mappings ${\textbf{H}}^{(s)}$.

### Methodology flowcharts

**Figure 1:**
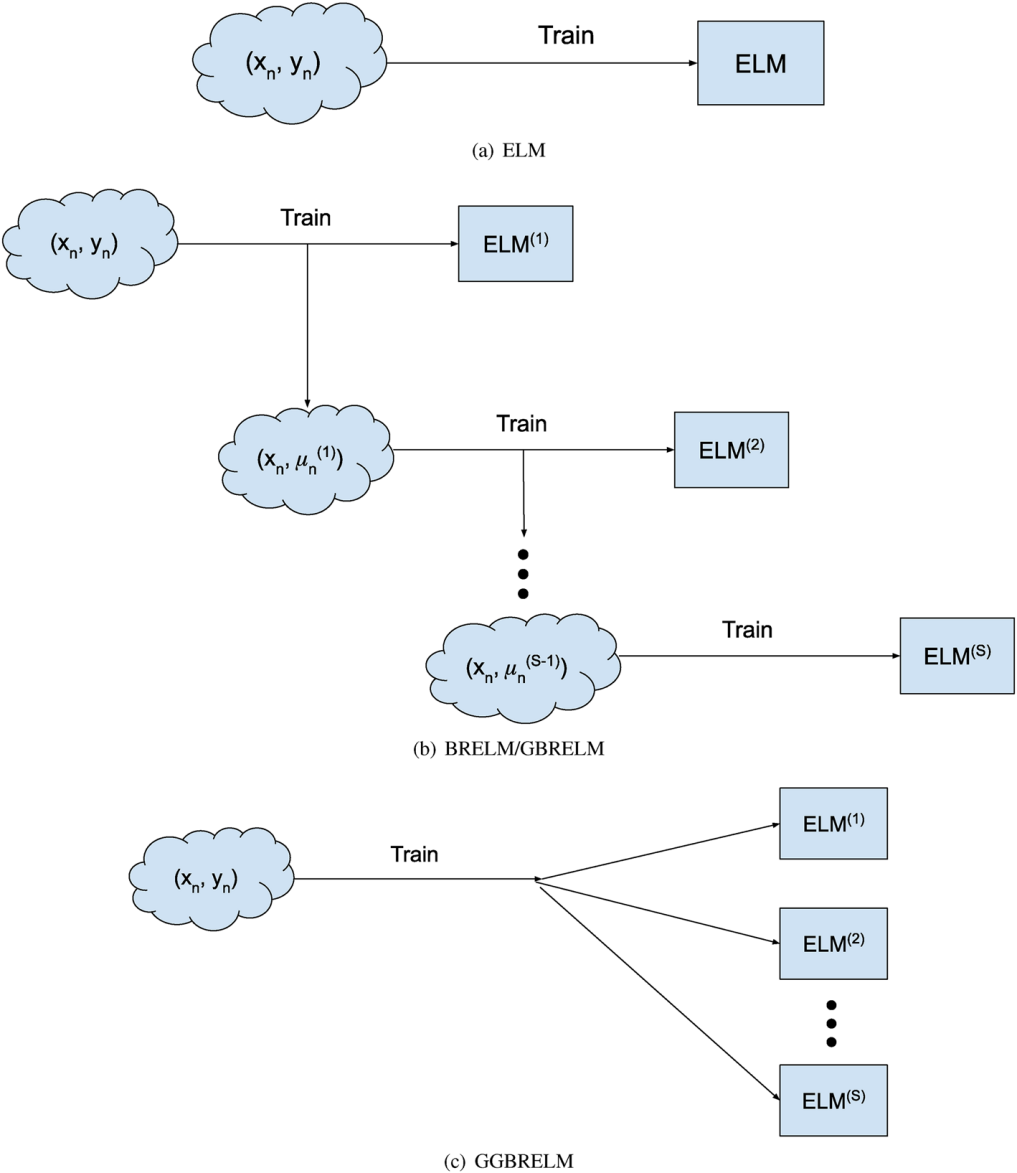
Minimalistic flowcharts of the different methodologies.

Figure 1 includes a graphical and minimalistic comparison of the methodologies involved in this paper. Note that ELM (a) trains one model in a single step, BRELM and GBRELM (b) train several models sequentially, and the proposed GGBRELM (c) trains all models in a single step, since BRELM, GBRELM and GGBRELM are ensemble methodologies.

### Analysis of the computational burden

The ELM model’s computational complexity is determined by the number of hidden nodes, denoted as *D*, the size of the training set, denoted as *N*, and the number of classes, *J*. To compute $\mathbf {H'H}$, it is needed to multiply a matrix of $D \times N$ by a $N \times D$ resulting in a complexity of $O(D \cdot N^2)$. Then, ELM must perform matrix inversion on a $D \times D$ matrix whose complexity is $O(D^{3})$ as shown in^46,47^. After that, a multiplication of the $\mathbf {H'Y}$, that is, $D \times N$ by $N \times J$ with a cost of $O(D \cdot N \cdot J)$. Finally, the resulting matrices $D \times D$ and $D \times J$ are multiplied with a computational time of $O(D^2 \cdot J)$. Therefore, the total computational complexity is $O(\text {ELM}) = O(D \cdot N^2 + D^{3} + D \cdot N \cdot J + D^2 \cdot J)$.

The computational cost for the BRELM and GBRELM methods also depends on the number of base learners *S*. Since these methodologies train *S* ELM models sequentially and each model is trained using the residual from the previous one as targets, the computational cost will be $O(S \cdot O(\text {ELM})+(S-1) (N\cdot D \cdot J))$.

Finally, considering that GGBRELM performs optimisation in a single step, the method must calculate a matrix inversion of a $DS \times DS$ matrix and multiply the result with a $DS \times NJ$ matrix. Given that the $\mathbf {H'H}$ matrix is symmetric, the computation of all the intermediate $\mathbf {H^{s'}H^{t}} \text { for } s={1, \ldots , S}, t={s, \ldots , S}$, a total of $S(S-1)/2$ multiplications of matrices $D \times N$ by $N \times D$ need to be performed, resulting in a complexity of $O(S(S-1)/2 \cdot D \cdot N^2)$. For this reason, the computational cost of GGBRELM is $O(S(S-1)/2 \cdot D \cdot N^2 + (DS)^{3}+ (DS)^2\cdot J+ DS \cdot N \cdot J)$.

## Experimental design

In order to evaluate the methodology presented in “Methodology of the proposal” Section, a comprehensive experimental environment has been implemented. In this sense, “Experiments” Section describes the experiments performed initially. “Datasets” Section includes a description of the datasets employed in the regression and classification problems. “Algorithms and parameters setting” Section contains a concise explanation of the algorithms selected for performing the comparative study and the set-up of their hyperparameters. Finally, the metrics implemented for the evaluation of the models are detailed in “Measures” Section, and the statistical tests carried out to validate the obtained results are defined in “Statistical tests” Section.

### Experiments

As stated before, the aim of this work is not only to improve the performance of the base learner (ELM) but also to overcome the disadvantages of the BRELM and, specifically, Generalised BRELM (GBRELM). Also, for comparison purposes, a recent kernel methodology is used (KBRELM, see Algorithms and parameters setting” Section). For this purpose, two experiments have been carried out:

- In the first experiment (E1), the number of neurons in the hidden layer was low. Thus, the smaller the number of hidden nodes, the worse ELM performs; on the other hand, GBRELM performs better.
- In the second experiment (E2), the number of nodes in the hidden layer is larger. Thereby, the performance capabilities of the ELM are high (strong learners), so this model achieves competitive results. At the same time, the GBRELM ensemble cannot take advantage of its ensemble architecture to improve its performance. As a classic ensemble, its performance increases when weak learners are used and decreases when complex learners are used.

In both experiments, the performance of the methodologies in the datasets will be analysed according to their size. Also, for the classification problems, the number of classes and the imbalance ratio, calculated as the ratio resulting from dividing the number of patterns of the majority class by the number of patterns of the minority class, will be examined.

The underlying idea is to demonstrate that GGBRELM outperforms ELM, GBRELM and KBRELM in both experimental scenarios by comparing them in regression and classification problems and performing an analysis according to different dataset properties.

### Datasets

Experimental validation has been performed on 71 classification datasets and 52 regression datasets, respectively. This selection was carried out to include in the reference datasets various types of classification/regression problems in terms of their field of application, their size (product of the number of patterns times the number of attributes), their number of classes, and their imbalanced ratio. Tables 1 and 2 show a summary of the main characteristics of the selected datasets: identification number (*ID*), which has been assigned by ordering the datasets from the highest to the lowest size, name (*Dataset*), number of instances (*#Inst.*), attributes (*#Attr.*) and size (*Size*). According to their size, databases have been divided into large (size > 100000), medium (10000 < size < 100000) and small (size < 10000). The number of classes (*#Classes*), their distribution (*Class distribution*) and the imbalanced ratio (*IR*) have also been included in the characterisation of the classification problem datasets (Table 1). Imbalanced datasets (IR > 2) have also been underlined for further analysis. From here to the end, the datasets are annotated according to their ID. While classification datasets are extracted from UCI Machine Learning Repository^48^, regression benchmark problems come from different machine learning repositories: UCI, Department of Statistics in the University of Florida^49^ and LIACC^50^.

**Table 1:** Characteristics of the selected classification datasets, sorted by size.

Classification datasets							
ID	Dataset	#Inst.	#Attr.	Size	#Classes	Class distribution	IR
Large datasets (Size > 100000)							
1	Weight-exercises	39242	53	2079826	5	(11159 7593 7214 6844 6432)	1.73
2	Cnae-9	1080	856	924480	9	(120 120 120 120 120 120 120 120 120)	1.00
3	Mushroom	8124	111	901764	2	(4208 3916)	1.07
4	Skin-segmentation	245057	3	735171	2	(194198 50859)	3.82
5	Statlog-shuttle	43500	9	391500	7	(34108 6748 2458 132 37 11 6)	5684.67
6	Letter-recognition	20000	16	320000	26	(813 805 803 796 792 789 787 786 783 783 775 773 768 766 764 761 758 755 753 752 748 747 739 736 734 734)	1.11
7	Spambase	4601	57	262257	2	(2788 1813)	1.54
8	Optical-recognition-handwritten-digits	3823	64	244672	10	(389 389 387 387 382 380 380 377 376 376)	1.03
9	Weight-lifting-exercises	4024	54	217296	5	(1370 1365 901 276 112)	12.23
10	Magic-gamma-telescope	19020	10	190200	2	(12332 6688)	1.84
11	Statlog-project-landsat-satellite	4435	36	159660	6	(1072 1038 961 479 470 415)	2.58
12	Ozone-level-detection-one	1848	72	133056	2	(1791 57)	31.42
13	Ozone-level-detection-eight	1847	72	132984	2	(1719 128)	13.43
14	Wall-following-robot-navigation-24	5456	24	130944	4	(2205 2097 826 328)	6.72
15	Chess-king-rook-vs-king-pawn	3196	38	121448	2	(1669 1527)	1.09
16	Electrical-grid	10000	12	120000	2	(6380 3620)	1.76
17	Pen-based-recognition-handwritten-digits	7494	16	119904	10	(780 780 780 779 778 720 720 719 719 719)	1.08
Medium datasets (10000 < Size < 100000)							
18	Thyroid-disease-ann-thyroid	3772	21	79212	3	(3488 191 93)	37.51
19	Thyroid-disease-allhyper	2800	27	75600	4	(2723 62 8 7)	389.00
20	Thyroid-disease-sick-euthyroid	3163	20	63260	2	(2870 293)	9.80
21	Hill-valley	606	100	60600	2	(305 301)	1.01
22	Hill-valley-noise	606	100	60600	2	(307 299)	1.03
23	Statlog-project-german-credit	1000	59	59000	2	(700 300)	2.33
24	Seismic-bumps	2584	22	56848	2	(2414 170)	14.20
25	Thyroid-disease-dis	2028	28	56784	2	(1989 39)	51.00
26	Thyroid-disease-sick	2028	28	56784	2	(1866 162)	11.52
27	Thyroid-disease-allbp	2028	23	46644	5	(936 716 265 82 29)	32.28
28	Qsar-biodegradation	1055	41	43255	2	(699 356)	1.96
29	Horse-colic	300	121	36300	2	(191 109)	1.75
30	Car-evaluation	1728	21	36288	4	(1210 384 69 65)	18.62
31	Libras-movement	360	90	32400	15	(24 24 24 24 24 24 24 24 24 24 24 24 24 24 24)	1.00
32	Credit-approval	666	46	30636	2	(367 299)	1.23
33	Tic-tac-toe-endgame	958	27	25866	2	(626 332)	1.89
34	Congressional-voting-records	435	48	20880	2	(267 168)	1.59
35	Breast-cancer-wisconsin-diagnostic	569	30	17070	2	(357 212)	1.68
36	Thoracic-surgery	470	27	12690	2	(400 70)	5.71
37	Connectionist-bench-sonar	208	60	12480	2	(111 97)	1.14
38	Dermatology	358	34	12172	6	(111 71 60 48 48 20)	5.55
39	Ionosphere	351	34	11934	2	(225 126)	1.79
40	Yeast	1484	8	11872	10	(463 429 244 163 51 44 35 30 20 5)	92.60
41	Breast-cancer	286	39	11154	2	(218 68)	3.21
42	Wall-following-robot-navigation-2	5456	2	10912	4	(2205 2097 826 328)	6.72
Small datasets (Size < 10000)							
43	Connectionist-bench	990	10	9900	11	(90 90 90 90 90 90 90 90 90 90 90)	1.00
44	Climate-model-simulation-crashes	540	18	9720	2	(494 46)	10.74
45	Teaching-assistant-evaluation	151	54	8154	3	(52 50 49)	1.06
46	Heart-disease-cleveland	299	23	6877	5	(161 54 36 35 13)	12.38
47	Breast-cancer-wisconsin-prognostic	194	32	6208	2	(148 46)	3.22
48	Breast-cancer-wisconsin	683	9	6147	2	(444 239)	1.86
49	Indian-liver-patient	579	10	5790	2	(414 165)	2.51
50	Heart-disease-hungarian	294	19	5586	2	(188 106)	1.77
51	Parkinsons	195	21	4095	2	(147 48)	3.06
52	Image-segmentation	210	19	3990	7	(30 30 30 30 30 30 30)	1.00
53	Spectf-heart	80	44	3520	2	(40 40)	1.00
54	Blood-transfusion-service-center	748	4	2992	2	(570 178)	3.20
55	Monks-problems-2	432	6	2592	2	(290 142)	2.04
56	Balance-scale	625	4	2500	3	(288 288 49)	5.88
57	Wine	178	13	2314	3	(71 59 48)	1.48
58	Planning-relax	182	12	2184	2	(130 52)	2.50
59	Soybean-small	47	45	2115	4	(17 10 10 10)	1.70
60	Glass-identification	214	9	1926	6	(76 70 29 17 13 9)	8.44
61	Hepatitis	80	19	1520	2	(67 13)	5.15
62	Seeds	210	7	1470	3	(70 70 70)	1.00
63	Thyroid-disease-new-thyroid	215	5	1075	3	(150 35 30)	5.00
64	Haberman-survival	306	3	918	2	(225 81)	2.78
65	Fertility	100	9	900	2	(88 12)	7.33
66	Monks-problems-1	124	6	744	2	(62 62)	1.00
67	Monks-problems-3	122	6	732	2	(62 60)	1.03
68	Balloons-a	20	4	80	2	(12 8)	1.50
69	Balloons-b	20	4	80	2	(12 8)	1.50
70	Balloons-c	20	4	80	2	(12 8)	1.50
71	Balloons-d	16	4	64	2	(9 7)	1.29

**Table 2:** Characteristics of the selected regression datasets, sorted by size.

Regression datasets					
ID	Dataset	#Inst.	#Attr.	Size	Repository
Large datasets (Size > 100000)					
1	News-popularity	39644	58	2299352	UCI ML repository
2	Casp	45730	9	411570	UCI ML repository
3	Friedman	40768	9	366912	LIACC (University of Porto)
4	Ailerons	7154	40	286160	LIACC (University of Porto)
5	Nwp-min	7590	21	159390	UCI ML repository
6	Elevators	8752	17	148784	LIACC regression repository
7	Electrical-grid	10000	12	120000	UCI ML repository
Medium datasets (10000 < Size < 100000)					
8	Parkinsons-motor	5875	16	94000	UCI ML repository
9	Parkinsons-total	5875	16	94000	UCI ML repository
10	Skillcraft	3338	18	60084	UCI ML repository
11	Winequality-white	4898	11	53878	UCI ML repository
12	Cpu-performance	209	245	51205	UCI ML repository
13	Abalone	4177	10	41770	UCI ML repository
14	Student-performance-por	649	43	27907	UCI ML repository
15	Parkinsons-speech	1040	26	27040	UCI ML repository
16	Usopen-men-2013a	126	168	21168	UCI ML repository
17	Usopen-men-2013b	126	168	21168	UCI ML repository
18	Frenchopen-men-2013a	123	170	20910	UCI ML repository
19	Frenchopen-men-2013b	123	170	20910	UCI ML repository
20	Wimbledon-women-2013a	118	170	20060	UCI ML repository
21	Wimbledon-women-2013b	118	170	20060	UCI ML repository
22	Wimbledon-men-2013a	113	163	18419	UCI ML repository
23	Wimbledon-men-2013b	113	163	18419	UCI ML repository
24	Winequality-red	1599	11	17589	UCI ML repository
25	Frenchopen-women-2013a	111	155	17205	UCI ML repository
26	Frenchopen-women-2013b	111	155	17205	UCI ML repository
27	Student-performance-mat	395	43	16985	UCI ML repository
28	Forestfires	517	28	14476	UCI ML repository
29	Ausopen-men-2013a	103	138	14214	UCI ML repository
30	Ausopen-men-2013b	103	138	14214	UCI ML repository
31	Ausopen-women-2013a	99	141	13959	UCI ML repository
32	Ausopen-women-2013b	99	141	13959	UCI ML repository
33	Triazines	186	60	11160	LIACC regression repository
Small datasets (Size < 10000)					
34	Automobile	160	62	9920	UCI ML repository
35	Usopen-women-2013a	74	106	7844	LIACC (University of Porto)
36	Airfoil-self-noise	1503	5	7515	LIACC (University of Porto)
37	Housing	506	13	6578	UCI ML repository
38	Auto-mpg	392	7	2744	UCI ML repository
39	Servo	167	12	2004	UCI ML repository
40	Pyrim	74	27	1998	UCI ML repository
41	Yatch	308	6	1848	UCI ML repository
42	Hybrid	153	11	1683	Departament of Statistics (University of Florida)
43	Lpga2009	146	11	1606	Departament of Statistics (University of Florida)
44	Brazilian-logistic	60	20	1200	UCI ML repository
45	Slump	103	7	721	UCI ML repository
46	Slump-flow	103	7	721	UCI ML repository
47	Slump-mpa	103	7	721	UCI ML repository
48	Japanemg	45	5	225	Departament of Statistics (University of Florida)
49	Beer	23	7	161	Departament of Statistics (University of Florida)
50	Const-maint	33	4	132	Departament of Statistics (University of Florida)
51	Diabetes	43	2	86	LIACC (University of Porto)
52	Texas-jan-temp	16	3	48	Departament of Statistics (University of Florida)

### Algorithms and parameters setting

The proposed method has been evaluated by comparing its results with respect to other recent state-of-the-art ELM proposals. The comparison methods are briefly described below:

- Extreme Learning Machine (ELM)^12^ (described in “Extreme learning machine” Section). In the model implementation, the weights and bias in the hidden layer were randomly generated following a uniform distribution. In contrast, the output weights were optimised using the ELM minimisation problem with $L_{2}$ regularisation.
- Generalised BRELM (GBRELM) (a version combining the algorithm described in “Boosting ridge extreme learning machine” Section with the generalisation of mapping functions ${\textbf{h}}^{(s)}$). This work compares the generalised version of Boosting Ridge for Extreme Learning Machine since it introduces variability into the model. Thus it would not make sense to compare with a simpler version where all ensemble elements have the same input layer.
- Generalised Global BRELM (GGBRELM) (described in Section “Methodology of the proposal”). The proposed methodology improves the sequential Generalised Boosting Ridge original architecture with a global approach.
- Kernel BRELM (KBRELM)^39^. In order to compare our proposal with a more recent methodology in the literature, we have also added a Boosting Ridge ensemble using as base learners Kernel Ridge Regression, as in^39^. This method works as the sequential Boosting Ridge for ELM presented in “Boosting ridge regression” Section but uses kernel trick instead of neural mapping. For it, Gaussian kernel was used, with hyperparameter $\gamma$,
  $$\begin{aligned} {\textbf{k}} (x_1, x_2) = \exp ^{ - \frac{{\Vert x_1 - x_2 \Vert }^2}{\gamma }}. \end{aligned}$$

The performance of the comparison methods depends critically on the setting of two hyperparameters: the regularisation parameter, *C*, and the number of hidden nodes, *D*. The hyperparameter *C* was determined by a grid search in a 5-fold nested cross-validation. The optimal value of the regularisation parameter for all comparison methods was determined with the following grid: $C \in \{ 10^{-2}, 10^{-1}, 1, 10, 10^2 \}$. The number of hidden nodes, *D*, in all models was set to $D = 10$ for the first experiment and $D = 1000$ for the second one. In the case of the KBRELM method, the $\gamma$ parameter needs to be crossvalidated, so it has been determined with the grid $\gamma \in \{ 10^{-2}, 10^{-1}, 1, 10, 10^2 \}$. The ensemble size for all the ensemble methods was set to 10 base learners.

The experimental results were obtained using a 10-fold cross-validation procedure, with 3 repetitions per fold. Thus, 30 error measures were obtained for all methods compared, ensuring adequate statistical significance of the results. The partitions were the same for all models compared. Input values were standardised, regression labels were scaled to [0, 1] and class labels were binarised, following “1-to-*J*” encoding^51^.

### Measures

The metrics used for performance validation were all standard metrics in their environments, that is, well-known and standard metrics for classification and regression problems. In this regard, the simplicity and success of applying the accuracy rate (*Acc*) have allowed it to be widely used as a performance measure for classification problems. However, the *Acc* is unsuitable for imbalanced datasets, which is one of the big tradeoffs when using the accuracy metric. As seen in Table 1, there are a total of 35 datasets with an *IR* higher than 2, which is the threshold value considered in this work. Therefore, it is more appropriate to use balanced accuracy ($Balanced\;Accuracy$), which is equal to the accuracy in balanced datasets and considers the imbalance of classes when it exists. In addition, two other classification metrics, Precision (*Precision*) and F-measure (*F*1), have also been used because they are useful in balanced and imbalanced scenarios.

Given a binary classification problem (positives and negatives patterns), it is considered:

- True positives (*TP*): positive patterns predicted as positive.
- False negatives (*FN*): positive patterns predicted as negative.
- False positives (*FP*): false patterns predicted as positive.
- True negative (*TN*): false patterns predicted as negative.

Then, these classification performance metrics are mathematically defined as follows:

- $Balanced\;Accuracy$ is the mean of Sensitivity and Specificity. Imbalanced datasets can be addressed by using the average of Sensitivity and Specificity. If a model only predicts accurately for the majority class in the dataset, it will receive a worse $Balanced\;Accuracy$ score:
  20 $$\begin{aligned} Balanced\;Accuracy = \frac{Sensivity + Specificity}{2} = \frac{1}{2}\left( \frac{TP}{TP+FN} + \frac{TN}{TN+FP} \right) . \end{aligned}$$
- *Precision* is the percentage of positive patterns predicted as positive with respect to the total of positive predicted patterns:
  21 $$\begin{aligned} Precision = \frac{TP}{TP+FP}. \end{aligned}$$
- *F*1 is the harmonic mean of the Precision and Recall:
  22 $$\begin{aligned} F1 = 2 \frac{Precision \cdot Recall}{Precision + Recall} = \frac{2TP}{2TP+FP+FN}. \end{aligned}$$

For multi-class problems, the metrics are calculated by comparing one class against all the others. The chosen class is considered positive, while the others are negative. This approach allows for obtaining a metric value for each of the classes. Then, the mean value is obtained.

The root mean square error (*RMSE*) and the determination coefficient ($R^2$) are the principal measures in the validation of an algorithm for regression problems:

- *RMSE* is the standard deviation of the differences between predicted and target values, and it is defined as:
  23 $$\begin{aligned} RMSE = \sqrt{\frac{1}{N} \sum _{n=1}^N \left( \hat{{\textbf{y}}}\left( {\textbf{x}}_{n}\right) - {y}_{n}\right) ^{2}}, \end{aligned}$$
  where $\hat{{\textbf{y}}}\left( {\textbf{x}}_{n}\right)$ is the predicted value for pattern ${\textbf{x}}_{n}$, and ${y}_{n}$, the real one.
- $R^2$ is the determination coefficient representing the proportion of the variation in the dependent variable that is predictable from the independent variables.
  24 $$\begin{aligned} R^2 = \frac{\sigma ^2_{\hat{{\textbf{y}}}, {\textbf{y}}}}{\sigma ^2_{\hat{{\textbf{y}}}} \sigma ^2_{{\textbf{y}}}}, \end{aligned}$$
  where ${\textbf{y}}$ and $\hat{{\textbf{y}}}$, are the real and predicted values, respectively.

### Statistical tests

In order to demonstrate that the GGBRELM model is a promising method in its field, it is crucial to validate its performance with respect to that of the comparison methods with statistical tests. For both experiments and for each metric, a pre-hoc test was applied with the evaluations of the methods on the different datasets to assess the statistical significance of the rank differences. For evaluations where the test detected statistical differences in method rankings, a post-hoc test was conducted to determine which models are distinctive among the multiple comparisons performed using the best performing method as the control method. For this purpose, nonparametric tests were applied. First, nonparametric Friedman’s tests^52^, with $Balanced\;Accuracy$, *Precision* and *F*1 (classification), and *RMSE* and $R^2$ (regression) ranking of the models as test variables, were carried out for $\alpha = 0.05$. Then, nonparametric Holm’s post-hoc test^53^ was implemented to determine whether the control method, the GGBRELM, statistically outperforms the comparison methods considering $\alpha = 0.05$ and taking into account each metric.

## Discussion of the results

This section includes the analysis of the experimental results obtained on the selected datasets. This part of the paper has been divided into two sections according to classification and regression datasets. For the sake of conciseness, it has been opted to provide only the relevant graphs and a summary of the statistical results.

### Classification datasets

**Figure 2:**
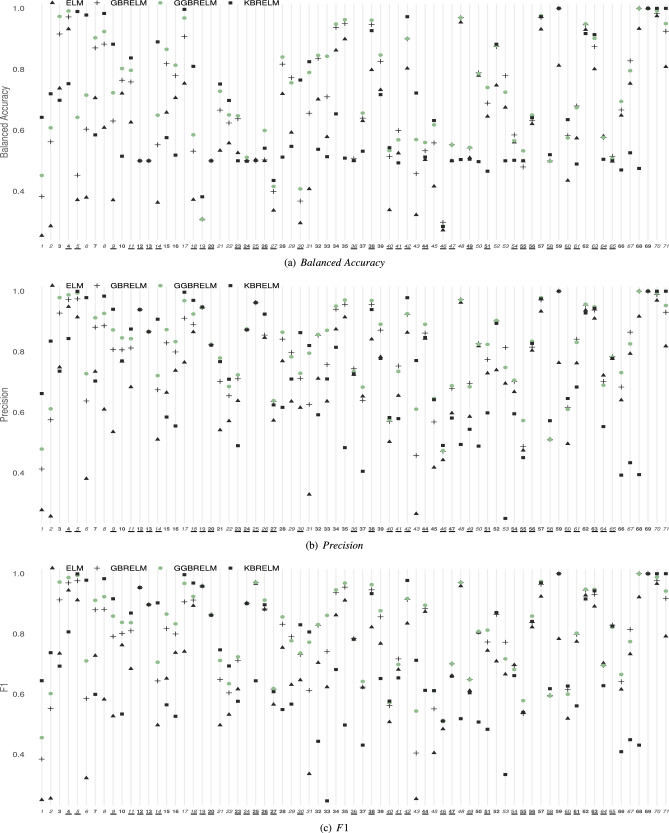
Performance plot on metrics for classification datasets using D = 10. The Y-axis represents the value of the metric, while the X-axis contains the IDs of the datasets sorted by size. If GGBRELM is the best for that dataset, its ID appears in bold, and if it is the second best, it appears in italics. Finally, imbalanced datasets are marked with an underline.

**Figure 3:**
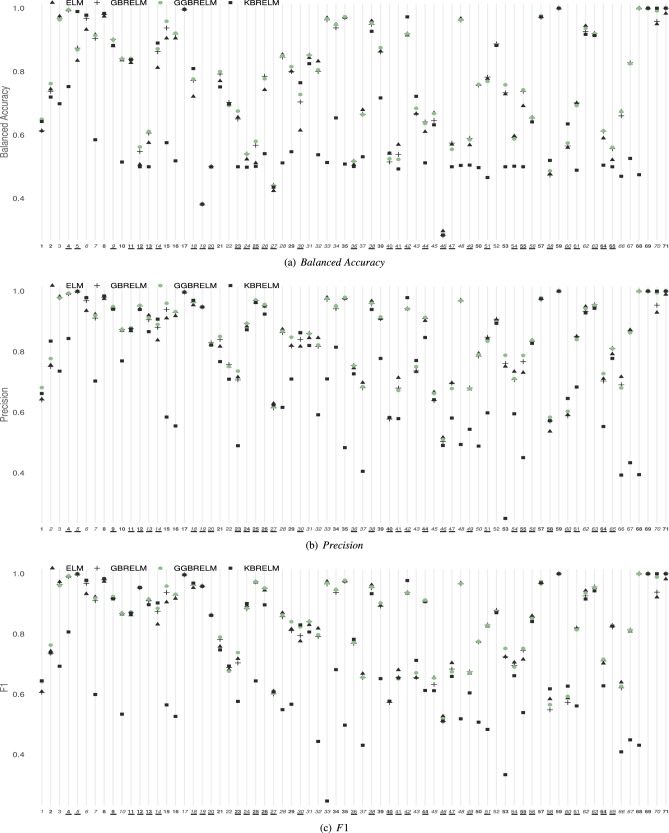
Performance plot on metrics for classification datasets using D = 1000. The Y-axis represents the value of the metric, while the X-axis contains the IDs of the datasets sorted by size. If GGBRELM is the best for that dataset, its ID appears in bold, and if it is the second best, it appears in italics. Finally, imbalanced datasets are marked with an underline.

The generalisation performances of the considered methods for E1 ($D=10$) and E2 ($D=1000$) in classification datasets are shown in Figs. 2 and 3, respectively ((a) $Balanced\;Accuracy$, (b) *Precision*, (c) *F*1). In those figures, the Y-axis represents the value of the reported metric, while the X-axis contains the IDs of the datasets sorted by size. If GGBRELM is the best for one dataset, its ID appears in bold, and if it is the second best, it appears in italics. Finally, imbalanced datasets are marked with an underline. For the case of the all classification metrics, the higher the point is located on the graph, the better performance of that method since the objective is to maximise these metrics.

As a general rule, it can be observed that the GGBRELM methodology outperforms the other approaches in $Balanced\;Accuracy$, *Precision* and *F*1 in both experiments. Significantly, the difference is greater in those datasets where all the methodologies do not achieve good performances.

In particular, in E1, when comparing $Balanced\;Accuracy$, GGBRELM performs better in 31 datasets, and it is the second best in 36, representing almost the total number of databases. For precision, it is the best in 36 datasets and the second one in 30. Moreover, for the F1, GGBRELM is also the best in 36 datasets and the second in 27. GBRELM and KBRELM have similar performance regarding the number of databases in which they are the best or second. ELM performance is lower than the ensemble approaches, according to the literature.

Furthermore, in experiment E2, where the classifiers are configured with a high number of neurons in the hidden layer, the ELM becomes more specialised. Hence its performance improves, and it should outperform the ensemble methods due to its disadvantages when using strong base learners, such as saturation or overfitting. Nevertheless, while it is true that GBRELM and KBRELM obtain worse results than ELM, GGBRELM overcomes this disadvantage of ensemble nature methods by getting more accurate results. Thus, GBBRELM achieves the best result in 27, 30 and 28 datasets in terms of $Balanced\;Accuracy$, *Precision* and *F*1, respectively, and the second best in 31, 30 and 30 datasets. Thus, the proposed methodology is also better than the three compared methods, as shown in Fig. 3.

**Table 3:** Results of the Friedman’s and Holm’s tests using GGBRELM as control algorithm (CA) when comparing its average $Balanced\;Accuracy$, *Precision* and *F*1 to those of ELM, GBRELM and KBRELM: corrected $\alpha$ values, compared methods and *p* values, all of them ordered by the number of comparison (i).

		Experiment 1		Experiment 2	
Balanced sccuracy	Friedman	$C_0 = (0, F_{0.05}=2.65)$	$F^*=27.80 (*)$	$C_0 = (0, F_{0.05}=2.65)$	$F^*=15 (*)$
	Holm	CA:GGBRELM		CA:GGBRELM	
i	$\alpha ^*_{0.05}$	Algorithm	$p_i$	Algorithm	$p_i$
1	0.017	ELM	0.0000 (*)	KBRELM	0.0000 (*)
2	0.025	KBRELM	0.0002 (*)	GBRELM	0.0063 (*)
3	0.050	GBRELM	0.0148 (*)	ELM	0.0375 (*)

Precision	Friedman	$C_0 = (0, F_{0.05}=2.65)$	$F^*=31.69 (*)$	$C_0 = (0, F_{0.05}=2.65)$	$F^*=10.76 (*)$
	Holm	CA:GGBRELM		CA:GGBRELM	
i	$\alpha ^*_{0.05}$	Algorithm	$p_i$	Algorithm	$p_i$
1	0.017	ELM	0.0000 (*)	KBRELM	0.0000 (*)
2	0.025	KBRELM	0.0015 (*)	GBRELM	0.0015 (*)
3	0.050	GBRELM	0.0085 (*)	ELM	0.0052 (*)

F1	Friedman	$C_0 = (0, F_{0.05}=2.65)$	$F^*=22.73 (*)$	$C_0 = (0, F_{0.05}=2.65)$	$F^*=9.89 (*)$
	Holm	CA:GGBRELM		CA:GGBRELM	
i	$\alpha ^*_{0.05}$	Algorithm	$p_i$	Algorithm	$p_i$
1	0.017	ELM	0.0000 (*)	KBRELM	0.0000 (*)
2	0.025	KBRELM	0.0000 (*)	GBRELM	0.0005 (*)
3	0.050	GBRELM	0.0177 (*)	ELM	0.0027 (*)

CA results statistically better than the compared algorithm are marked with (*).

As mentioned above, a set of statistical tests have been carried out to analyse the results from statistical hypothesis contrasts, summarising the results in Table 3. For the Friedman’s tests and a level of significance $\alpha = 5\%$, the confidence interval is $C_0 = (0, F_{0.05} = 2.65)$, and the F-distribution statistical value considering $Balanced\;Accuracy$ rankings is $F^* = 27.80$, considering *Precision* rankings is $F^* = 31.69$ and taking into account *F*1 is $F^* = 22.73$ in the experiment E1 (D = 10), while in the E2 experiment (D = 1000), $F^* = 15$, $F^* = 10.76$ and $F^* = 9.89$, respectively. Consequently, in both experiments, the test rejects the null-hypothesis stating that all algorithms perform equally in mean ranking of $Balanced\;Accuracy$, *Precision* and *F*1. That is, the algorithm effect is statistically significant. For this reason, it is considered the best performing method as a control method for a post-hoc test, comparing this algorithm with the rest of the methods. In this way, Table 3 also shows the results of Holm’s test. When using GGBRELM as the control algorithm (CA), Holm’s test shows that $p_i < \alpha ^*_i$ in all cases, for $\alpha =0.05$, confirming that there are statistically significant differences favouring GGBRELM in both experiments and for each metric.

#### Discussion considering dataset size

As aforementioned, the datasets have been sorted in decreasing order of size and have been divided into three categories according to it, as shown in Table 1: 17 large datasets (*IDs* 1-17), 25 medium (*IDs* 18-42) and 29 small ones (*IDs* 43-71).

Looking at E1, for large datasets, GGBRELM is the best in 8 datasets and the second in the remaining ones for all metrics. It is the best in 12, 13 and 13 medium datasets and the second in 11, 10 and 9 according to $Balanced\;Accuracy$, *Precision* and *F*1, respectively. For small datasets, the best results are achieved on 11, 15 and 15, and the second best on 16, 11 and 9 datasets, depending on the metric analysed.

For the case of E2, for large datasets, GGBRELM is the best in 11, 10 and 9 and the second best in 4, 6 and 7. For medium datasets, the best are obtained in 6, 10 and 9, while the second best results are achieved in 14, 11 and 10. Finally, the best results are obtained in 10 and the second best in 13 small datasets in all metrics.

As can be seen, regardless of size, the GGBRELM method performs quite well. However, for both E1 and E2, the best results are concentrated in the large datasets being the best or second best method in almost all metrics in both experiments. In the smallest datasets, the improvement of the proposal is not as noticeable as in the remaining ones. It makes sense since they are databases without difficulty and are easier to solve by any method.

#### Discussion considering imbalanced datasets

In the experimental validation, there are a total of 35 imbalanced datasets. As stated, for each classification database, the *IR* has been calculated as the ratio of the number of patterns in the majority class to the number of patterns in the minority class. The *IR* has been reported in Table 1, underlining those datasets with an $IR>2$. In addition, in Figs. 2 and 3, the *IDs* of these imbalanced datasets have also been underlined, making it easier to discuss the results by taking them into account.

Considering the first experiment with *D* set to 10, GGBRELM achieves the best result on 13 datasets and the second best on 18, resulting in almost the total number of databases, considering the $Balanced\;Accuracy$ metric. Similar is what happens with the other two metrics, being the best in 15 and second best in 15 for *Precision* and obtaining the best results in 16 and second best in 11 with *F*1. In this case, it is worth noting that the second method would be GBRELM on average for the three metrics. Although KBRELM obtains the best result in many databases, this showed an unstable behaviour since it is either very good or the worst, depending on the dataset.

As for E2, the same happens for GGBRELM, being the best method for the three metrics in 9, 13 and 12 datasets, respectively, and the second best method in 18, 16 and 13. It is important to note that for imbalanced datasets, the GBRELM method has approximately the same average performance in all metrics with respect to ELM, but ELM is still slightly better than GBRELM.

From this analysis, it can be concluded that the proposed GGBRELM method not only performs well on all metrics for all databases but is also the most appropriate for imbalanced datasets.

#### Discussion considering the number of classes

From column *#Classes* in Table 1 and Figs. 2 and 3, the influence of the number of classes on the results obtained can be analysed.

Thus, for E1 and the 44 binary problems, GGBRELM is the best algorithm on average since it is the best on 26, 27 and 28 databases depending on the analysed metric ($Balanced\;Accuracy$, *Precision* and *F*1). In addition, it is the second best on 16, 14 and 11, respectively. In the case of multiclass problems, and specifically as the number of classes increases, KBRELM performs similarly to GGBRELM in this experiment. This can be explained by the fact that the higher the number of classes, the more complex the problem becomes, and the algorithms with a higher number of connections benefit, as is the case of kernels.

However, for the case of E2, i.e., when GGBRELM is provided with more neurons in its base classifiers, the results indicate that it performs better on average than the rest of the algorithms in binary and multiclass problems in all metrics. Thus, in binary problems, GGBRELM is the best in 20, 22 and 21, and the second in 14, 13 and 13, respectively. For the case of problems with a more significant number of classes, it is the best in 7, 8 and 7 and the second best in practically the remaining ones, making it the best algorithm on average.

### Regression datasets

**Figure 4:**
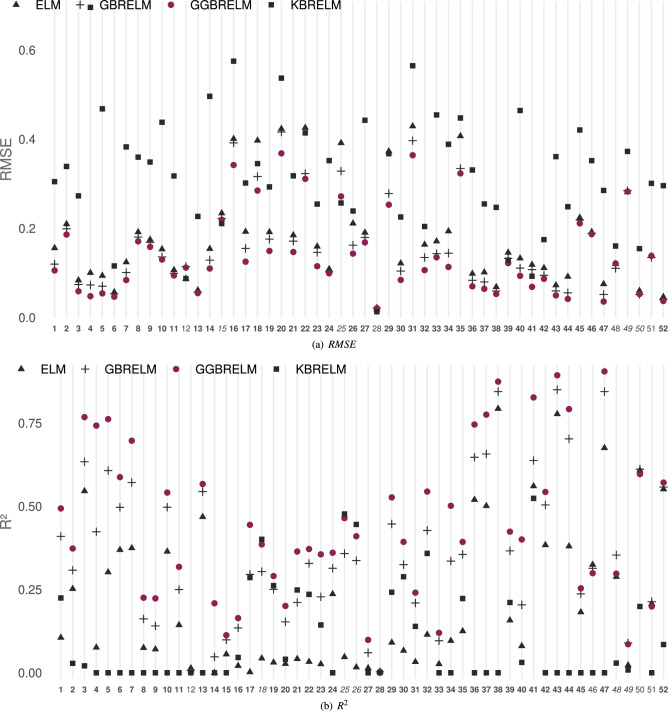
Performance plot on metrics for regression datasets using D = 10. The Y-axis represents the value of the metric, while the X-axis contains the IDs of the datasets sorted by size. If GGBRELM is the best for that dataset, its ID appears in bold, and if it is the second best, it appears in italics.

**Figure 5:**
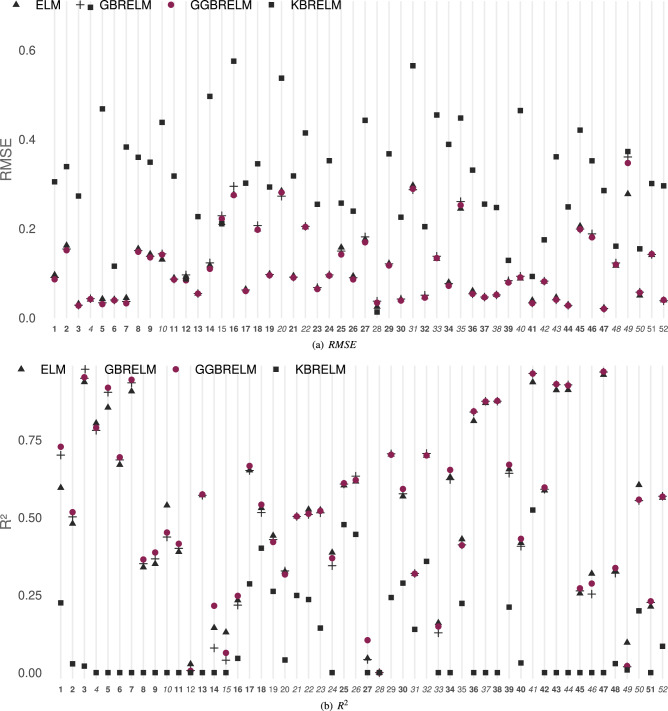
Performance plot on metrics for regression datasets using D = 1000. The Y-axis represents the value of the metric, while the X-axis contains the IDs of the datasets sorted by size. If GGBRELM is the best for that dataset, its ID appears in bold, and if it is the second best, it appears in italics.

The performances of the considered methods for E1 ($D=10$) and E2 ($D=1000$) in regression datasets are shown in Figs. 4 and 5, respectively ((a) *RMSE*, (b) $R^2$). As in classification datasets, the Y-axis represents the value of the reported metric, while the X-axis contains the IDs of the datasets sorted by size. If GGBRELM is the best for one dataset, its ID appears in bold, and if it is the second best, it appears in italics. For the case of the *RMSE* metric, the lower the point is located on the graph, the better performance of that method since the objective is to minimise this metric. The opposite occurs in the $R^2$ metric because it must be maximised.

The findings unambiguously demonstrate that the GGBRELM methodology outperforms the alternative approaches in both experiments and across both metrics. This distinction is especially evident in datasets where the other methodologies exhibit suboptimal performance.

Thus, in the case of E1, GGBRELM is the best method in 44 datasets and the second best in 4 datasets in terms of *RMSE*. In addition, it is the best method in 43 datasets and the second best in 5 datasets when comparing $R^2$. With a low number of neurons, GBRELM also outperforms ELM since it is a weak learner. However, KBRELM does not seem to perform well in problems of this nature, being the worst regressor of the four methods.

Furthermore, in experiment E2, GGBRELM overcomes the disadvantage of ensemble nature methods by getting more accurate results regarding *RMSE* and $R^2$. Hence, GGBRELM achieves the better *RMSE* performance in 34 datasets and the second best in 14. Similarly, it gets the best $R^2$ in 28 datasets and the second best in 19.

In the same way, as in classification datasets, four Friedman’s tests have been run showing the rejection of the null-hypothesis given that, for $\alpha = 5\%$, the confidence interval is $C_0 = (0, F_{0.05} = 2.66)$, and the statistical values for *RMSE* and $R^2$ are $F^* = 102.63$ and $F^* = 101.97$ in E1, and $F^* = 77.21$ and $F^* = 91.05$ in E2 (Table 4). This Table also shows the results of Holm’s test comparing *RMSE* and $R^2$. Again, when using GGBRELM as the control algorithm (CA), Holm’s test shows that $p_i < \alpha ^*_i$ in all cases, for $\alpha =0.05$, confirming that there are statistically significant differences favouring GGBRELM in both experiments and metrics.

**Table 4:** Results of the Friedman’s and Holm’s tests using GGBRELM as control algorithm (CA) when comparing its average *RMSE* and $R^2$ to those of ELM, GBRELM and KBRELM: corrected $\alpha$ values, compared methods and *p*-values, all of them ordered by the number of comparison (i). CA results statistically better than the compared algorithm are marked with (*).

		Experiment 1		Experiment 2	
RMSE	Friedman	$C_0 = (0, F_{0.05}=2.66)$	$F^*=102.63 (*)$	$C_0 = (0, F_{0.05}=2.66)$	$F^*=77.21 (*)$
	Holm	CA:GGBRELM		CA:GGBRELM	
i	$\alpha ^*_{0.05}$	Algorithm	$p_i$	Algorithm	$p_i$
1	0.017	KBRELM	0.0000 (*)	KBRELM	0.0000 (*)
2	0.025	ELM	0.0000 (*)	GBRELM	0.0001 (*)
3	0.050	GBRELM	0.0005 (*)	ELM	0.0004 (*)

$\mathbf {R^2}$	Friedman	$C_0 = (0, F_{0.05}=2.66)$	$F^*=101.97 (*)$	$C_0 = (0, F_{0.05}=2.66)$	$F^*=91.05 (*)$
	Holm	CA:GGBRELM		CA:GGBRELM	
i	$\alpha ^*_{0.05}$	Algorithm	$p_i$	Algorithm	$p_i$
1	0.017	KBRELM	0.0000 (*)	KBRELM	0.0000 (*)
2	0.025	ELM	0.0000 (*)	GBRELM	0.0063 (*)
3	0.050	GBRELM	0.0024 (*)	ELM	0.0185 (*)

#### Discussion considering dataset size

The regression datasets have been ordered from the highest to the smallest size and have also been divided into three categories as shown in Table 2: 7 large datasets (*IDs* 1-7), 26 medium (*ID* 8-33) and 29 small (*ID* 34-52).

Considering E1, for large datasets, GGBRELM is the best in all datasets for all metrics. For medium size, it is the best in 22 in both metrics and the second in 2 and 3, respectively. For small datasets, the best results are achieved on 15 and 14, and the second best on 2 datasets in both metrics.

For the case of E2, for large datasets, GGBRELM is the best in 6 datasets and the second in 1 for both metrics. For medium datasets, the best are obtained in 19 and 11, while the second best results are obtained in 5 and 11. Finally, for small datasets, the best are obtained in 9 and 11, and the second best in 8 and 7 datasets.

In both experiments, the dataset size does not influence since, in all cases, the GGBRELM algorithm is much better than the others. However, it can be observed how in the five smallest databases, the performance difference of GGBRELM with respect to the other methods decreases since they lack complexity and are susceptible to being solved with any method.

## Conclusions

This paper presents a new ensemble methodology that tackles the problem of base learners saturation and a drop in performance when strong base learners are used in the ensemble method, avoiding increase iteratively the size of the ensemble. To solve this, this method performs a global optimisation in the Boosting Ridge methodology, using Extreme Learning Machine models as base learners. The proposed ensemble method, Generalised Global Boosting Ridge for Extreme Learning Machine, generates a set of initial input layer mappings with different parameters for their hidden layers. The output layer weights are optimised in one step, reducing the generalisation error of the ensemble.

A complete experimentation has been carried out, taking into account 71 classification datasets, analysing their size, the number of classes and the imbalance ratio, and 52 regression datasets considering their size, all from different application domains. The experiments show that i) the proposed Generalised Global ensemble method for ELM outperforms Generalised Boosting Ridge in different contexts, that is, low number and high number of neurons, and ii) Generalised Global methodology improves the results of ELM when it is specialised with a high number of neurons, overcoming the disadvantage of ensemble methods in these scenarios. Instead of relying on generating diversity through weak learners (low number of neurons), our method depends on its optimisation in the final prediction of the ensemble as a whole, thus not relying on the implicit diversity of the hidden neurons mapping.

In future work, it planned to adapt the ensemble learning framework to other base learners and other machine learning paradigms, such as ordinal regression or semisupervised learning. And finally, the application of the methodology to real-world problems could be proposed.

## Data Availability

The databases used together with the code necessary for their extraction are available at https://github.com/cperales/uci-download-process. The code generated in the experimental design, including the proposed methodology is available at https://github.com/cperales/pyridge. The whole table results obtained during the current study are available from the corresponding author upon reasonable request.
